# Victim Selection Patterns in Sexual Offenders With Severe Mental Illness: An Exploratory Forensic Evaluation Study

**DOI:** 10.31083/AP50083

**Published:** 2026-06-28

**Authors:** Chia-Heng Lin, Wen-Ching Hsieh, Li-Ting Lin, Chia-Hsiang Chan

**Affiliations:** ^1^Department of General Psychiatry, Bali Psychiatric Center, Ministry of Health and Welfare, 249 New Taipei, Taiwan; ^2^Department of Psychology, Taoyuan Psychiatric Center, Ministry of Health and Welfare, 330 Taoyuan, Taiwan; ^3^Department of General Psychiatry, Taoyuan Psychiatric Center, Ministry of Health and Welfare, 330 Taoyuan, Taiwan; ^4^Institute of Brain Science, National Yang Ming Chiao Tung University, 112 Taipei, Taiwan; ^5^Department of Psychology, Chung Yuan Christian University, 320 Taoyuan, Taiwan

**Keywords:** sexual offenders, severe mental illness, schizophrenia spectrum disorder, victim, bipolar disorder

## Abstract

**Background:**

Sexual offenders with severe mental illness (SMI) remain underexamined, particularly regarding victim-selection patterns that may inform risk assessment and management. This study compared victim-related and selected clinical characteristics between sexual offenders with and without SMI in a court-referred forensic psychiatric evaluation sample.

**Methods:**

This retrospective study included 86 male sexual offenders referred for forensic psychiatric evaluation between 2009 and 2022. Participants were categorized into an SMI group (n = 21; schizophrenia-spectrum/other psychotic disorders or bipolar-related disorders) and a non-SMI group (n = 65). Group comparisons were conducted using bivariate analyses, and exploratory logistic regression examined victim-related correlates of SMI status.

**Results:**

Compared with non-SMI offenders, offenders with SMI were more likely to have adult victims and stranger victims. In the final model, adult victim (odds ratio (OR) = 4.40, 95% confidence interval (CI) 1.30–14.91) and stranger victim (OR = 3.81, 95% CI 1.16–12.46) remained associated with SMI status. Substance use disorder was more common in the non-SMI group.

**Conclusions:**

In this exploratory forensic sample, sexual offenders with SMI showed distinct victim-selection patterns. Because the SMI subgroup was small and consisted predominantly of schizophrenia-spectrum disorders, these findings should be interpreted cautiously and regarded as preliminary.

## Main Points

1. Sexual offenders with severe mental illness (SMI) showed distinct victim-selection patterns, with substantially higher odds of offending against adult victims (odds ratio (OR) = 4.40, 95% confidence interval (CI) 1.30–14.91) and strangers (OR = 3.81, 95% CI 1.16–12.46) compared with offenders without SMI.

2. Group differences were driven more by victim-related factors than by general background risk markers: offenders with and without SMI did not differ significantly in sociodemographic characteristics or prior criminal history in this forensic evaluation sample.

3. Sexual offending among individuals with SMI in this sample was less linked to substance-use comorbidity and may involve different criminogenic pathways.

## 1. Introduction

Public perception, often shaped by media narratives rather than empirical evidence, tends to characterize sexual offenders as a homogeneous, high-risk group [1,2]. A Swedish study comparing 8495 male sex offenders with a control group of 19,935 non-offenders found that sex offenders were more likely to have severe mental illnesses (SMIs), such as schizophrenia (odds ratio (OR) = 4.8, 95% confidence interval (CI) = 3.4–6.7), and bipolar disorder (OR = 3.4, 95% CI = 1.8–6.4). Among the included sex offenders, approximately 20% had a history of psychiatric hospitalization. Risk factors for sexual offending within this group included a prior history of sexual offenses, younger age, deviant sexual preferences, and interpersonal relationship problems [3].

According to official local crime statistics, the rate of sexual offenses has not significantly increased over the past decade. Most sex offenders were already acquainted with their victims. Additionally, approximately half of the victims are adults [4,5]. However, domestic victim-focused evidence regarding sexual offenders with SMIs remains critically underexamined. Understanding whether SMI is associated with distinct victim-selection patterns may contribute to future forensic research and hypothesis generation in this area.

Accordingly, the present study sought to compare demographic, clinical, judicial, and victim-related characteristics between local sexual offenders with SMI and those without SMI. Identifying sexual offenders with SMI in general correctional settings is logistically and ethically challenging [6]; therefore, this study will use sex offenders referred by courts or prosecutors to psychiatric teams for forensic mental health evaluations as the sample to explore whether sexual offenders with SMI differ from those without SMI in demographic, clinical, judicial, and victim-related characteristics in a forensic evaluation sample.

## 2. Materials and Methods

### 2.1 Study Design and Setting

This retrospective study was conducted in Taoyuan Psychiatric Center under the Ministry of Health and Welfare that provides forensic psychiatric evaluations for courts and prosecutors. The study period covered evaluations conducted between October 2009 and February 2022.

### 2.2 Participants and Group Definition

The study included sexual offenders referred by judicial authorities for forensic psychiatric evaluation. Charges and psychiatric diagnoses were extracted from multidisciplinary forensic evaluation reports. Mental illnesses are defined based on the diagnostic criteria listed in the latest edition of the Diagnostic and Statistical Manual of Mental Disorders, Fifth Edition, Text Revision (DSM-5-TR) [7]. SMI was operationally defined as schizophrenia-spectrum and other psychotic disorders or bipolar and related disorders [8,9]. Offenders without these diagnoses were classified as non-SMI. According to the National Institute of Justice in the U.S. [10], sex-related offenses are defined as any criminal acts involving a sexual component, including those involving actual or threatened physical harm (e.g., sexual assault) or causing psychological or emotional harm to victims (e.g., child pornography).

### 2.3 Measures

Variables extracted from the reports included sociodemographic characteristics (age at the time of the offense, biological sex, educational level, employment status, and marital status), selected clinical variables, prior criminal history, and victim-related characteristics. Offenders were categorized as having poor treatment adherence if the frequency and duration of their medication or non-medication treatments were less than 80% of the prescribed regimen prior to the offense [11]. Following the U.S. Centers for Disease Control (CDC) definition, this study categorizes any history of violence, abuse, neglect, or household dysfunction in sex offenders’ backgrounds as “Adverse Childhood Experiences (ACEs)” [12]. Based on the 2022 Crime Status and Analysis of the Republic of China [4] and the types of violent crimes included in the FBI’s Uniform Crime Reporting (UCR) Program [13], this study defines violent crimes as charges related to murder, attempted murder, aggravated assault, assault, offenses against sexual autonomy, robbery, mugging, intimidation, unlawful detention, domestic violence, and arson.

Victim-related variables included victim age category (minor vs. adult), victim-offender relationship (familial, acquainted, or stranger), repeated victimization, victim sex, and offense location. Because the study relied on retrospective forensic reports prepared for clinical-legal rather than research purposes, variable completeness and detail were not uniform across cases.

### 2.4 Statistical Analysis

Continuous variables were compared using independent-samples *t*-tests and categorical variables using chi-square tests. Cramer’s V for chi-square tests and Cohen’s d for *t*-tests were calculated. Given the limited number of offenders with SMI, the multivariable analysis was designed as an exploratory and parsimonious logistic regression model restricted to victim-related variables that differed in bivariate analyses. ORs and 95% CIs are reported. Model fit was evaluated using Cox & Snell R^2^ and Nagelkerke R^2^. Multicollinearity among predictors was assessed using variance inflation factors (VIF). All statistical analyses were two-tailed, and a *p*-value < 0.05 was considered statistically significant.

## 3. Results

A total of 722 forensic psychiatric evaluation cases were initially screened. After exclusion of cases with poor documentation and cases unrelated to sexual offending, the final sample consisted of 86 male sexual offenders (Table 1). Of these, 21 were classified as having SMI and 65 as not having SMI. Within the SMI subgroup, 19 of 21 cases involved schizophrenia-spectrum or other psychotic disorders, and 2 involved bipolar and related disorders.

**Table 1.** **Comparison of demographic characteristics and clinical factors between sexual offenders with and without SMIs**.

		With SMIs (n = 21)		Without SMIs (n = 65)				
		Mean	SD	Mean	SD	t	Effect size (d/V)	*p*
Age		34.43	15.57	37.72	14.35	–0.90	0.22	0.37
		n	%	n	%	χ^2^		*p*
Male		21	100	65	100			
Marital status								
	Single	19	90.5	51	78.5	1.51	0.13	0.34
	Married	2	9.5	13	20.0	1.21	0.12	0.34
	Separated	0	0	1	1.5	0.33	0.06	1.00
Unemployed		18	85.7	55	84.6	0.02	0.01	1.00
Below compulsory educational level		3	14.3	17	26.2	1.25	0.12	0.38
History of suicide attempts		2	9.5	1	1.5	3.00	0.19	0.15
Poor treatment compliance		16	76.2	60	92.3	4.01	0.22	0.06
Adverse childhood experiences		6	28.6	10	15.4	1.82	0.15	0.20

SD, standard deviation; SMI, severe mental illness.

No significant between-group differences were observed in age, marital status, employment, or educational level. Prior criminal history and recorded violent crime history also did not differ significantly between groups (Table 2). Among clinical variables, substance use disorder was more common in the non-SMI group.

**Table 2.** **Comparison of forensic and victim-related characteristics between sexual offenders with and without SMIs**.

		With SMIs (n = 21)		Without SMIs (n = 65)				
		n	%	n	%	χ^2^	Effect size (V)	*p*
Criminal history								
	Attempted murder	1	4.8	1	1.5	0.73	0.09	0.43
	Assault	1	4.8	3	4.6	0.00	0.00	1.00
	Sexual offense	3	14.3	9	13.8	0.00	0.00	1.00
	Illicit substance use	0	0	5	7.7	1.72	0.14	0.33
Recorded previous violent crimes		5	23.8	12	18.5	0.27	0.06	0.75
Victim Characteristics								
	Female	20	95.2	62	95.4	0.00	0.00	1.00
	Minors	5	23.8	45	69.2	13.46	0.40	<0.01
	Adults	16	76.2	20	30.8	13.46	0.40	<0.01
Previous victimization		0	0	2	3.1	0.66	0.09	1.00
Relationship with offender								
	Family member	1	4.8	26	40.0	9.15	0.33	<0.01
	Non-family but known	5	23.8	21	32.3	0.54	0.08	0.60
	Stranger	15	71.4	18	27.7	12.84	0.39	<0.01
Location of offense								
	Private residence	6	28.6	36	55.4	4.57	0.23	0.05
	Outdoor/concealed area	15	71.4	26	40.0	6.29	0.27	0.02
	Hotel	0	0	3	4.6	1.00	0.11	1.00

Victim-related differences were more pronounced. Compared with offenders without SMI, offenders with SMI were more likely to offend against adult victims (76.2% vs. 30.8%) and stranger victims (71.4% vs. 27.7%), and less likely to offend against familial victims. Offenses committed by offenders with SMI were also more likely to occur in outdoor or secluded areas in bivariate analyses.

In the exploratory logistic regression model (Table 3), adult victim and stranger victim remained independently associated with SMI status. The odds of SMI were higher in cases involving adult victims (OR = 4.40, 95% CI 1.30–14.91) and stranger victims (OR = 3.81, 95% CI 1.16–12.46). Offense location did not retain additional explanatory value once these victim-related variables were included.

**Table 3.** **Victim-related factors associated with SMI status**.

	Sexual offenders with SMIs		
	OR	95% CI	*p*
The victim is an adult	4.40	1.30–14.91	0.02
The victim is a stranger	3.81	1.16–12.46	0.03

Note: Final model adjusted for victim-related variables identified in bivariate analyses, including adult victim and stranger victim. Model fit indices: Cox & Snell R^2^ = 0.20; Nagelkerke R^2^ = 0.29; Hosmer–Lemeshow test *p* = 0.62. OR, odds ratio; CI, confidence interval.

## 4. Discussion

The findings indicated that, compared with offenders without SMI, offenders with SMI were more likely to have adult and stranger victims, and were more likely to occur in outdoor or concealed areas in bivariate analyses. In this sample, offenders with SMI were less likely than offenders without SMI to offend against minors or familial victims.

The pattern observed may be consistent with the possibility that social isolation and situational opportunity contribute to victim selection in some offenders with SMI. Specifically, severe mental illness often emerges in early adulthood—a developmental period in which sexual needs and social-cue maturation are still in progress. The presence of SMI may disrupt social-cue perception, weaken self-regulatory capacities, and generate profound feelings of isolation, loneliness, and demoralization. These factors, in turn, may contribute to criminogenic pathways involving inappropriate sexual behaviors [14]. In an Israeli incarcerated sample [15], compared with schizophrenic non-sexual offenders, offenders with schizophrenia demonstrated a greater tendency to target female victims. Among psychotic male offenders, the motivation underlying sexual offenses against women may be primarily driven by sexual arousal, misinterpretation of social cues, or situational opportunity [16].

### Limitations

Several limitations should be noted. First, the study was based on a single forensic service and included only court-referred offenders, limiting generalizability. Second, the SMI subgroup was small, which reduced statistical power and yielded wide confidence intervals. Also, the SMI subgroup consisted predominantly of schizophrenia-spectrum and other psychotic disorders, with only two cases involving bipolar-related disorders. Accordingly, the results pertain more to schizophrenia-spectrum disorders than to SMI broadly. Third, all variables were retrospectively extracted from forensic reports rather than collected through a standardized research protocol, and inter-rater reliability for coding was not formally assessed. Fourth, diagnoses were based on clinical forensic evaluations rather than structured diagnostic instruments. Finally, the exploratory nature of the regression modeling requires caution in interpretation. These findings should therefore be viewed as preliminary and hypothesis-generating rather than practice-defining.

## 5. Conclusions

In this exploratory forensic evaluation sample, sexual offenders with SMI were more likely than offenders without SMI to target adult and stranger victims. Because the SMI subgroup was small and composed predominantly of schizophrenia-spectrum disorders, the findings should be interpreted cautiously. Larger studies with more diagnostically diverse samples are needed to determine whether these victim-selection patterns are replicable and clinically meaningful.

## Data Availability

Upon request, and subject to review, the authors will provide data that support the findings of this study.
